# Nonselective Bottlenecks Control the Divergence and Diversification of Phase-Variable Bacterial Populations

**DOI:** 10.1128/mBio.02311-16

**Published:** 2017-04-04

**Authors:** Jack Aidley, Shweta Rajopadhye, Nwanekka M. Akinyemi, Lea Lango-Scholey, Christopher D. Bayliss

**Affiliations:** Department of Genetics, University of Leicester, Leicester, United Kingdom; University of York; Institut Pasteur

## Abstract

Phase variation occurs in many pathogenic and commensal bacteria and is a major generator of genetic variability. A putative advantage of phase variation is to counter reductions in variability imposed by nonselective bottlenecks during transmission. Genomes of *Campylobacter jejuni*, a widespread food-borne pathogen, contain multiple phase-variable loci whose rapid, stochastic variation is generated by hypermutable simple sequence repeat tracts. These loci can occupy a vast number of combinatorial expression states (phasotypes) enabling populations to rapidly access phenotypic diversity. The imposition of nonselective bottlenecks can perturb the relative frequencies of phasotypes, changing both within-population diversity and divergence from the initial population. Using both *in vitro* testing of *C. jejuni* populations and a simple stochastic simulation of phasotype change, we observed that single-cell bottlenecks produce output populations of low diversity but with bimodal patterns of either high or low divergence. Conversely, large bottlenecks allow divergence only by accumulation of diversity, while interpolation between these extremes is observed in intermediary bottlenecks. These patterns are sensitive to the genetic diversity of initial populations but stable over a range of mutation rates and number of loci. The qualitative similarities of experimental and *in silico* modeling indicate that the observed patterns are robust and applicable to other systems where localized hypermutation is a defining feature. We conclude that while phase variation will maintain bacterial population diversity in the face of intermediate bottlenecks, narrow transmission-associated bottlenecks could produce host-to-host variation in bacterial phenotypes and hence stochastic variation in colonization and disease outcomes.

## IMPORTANCE

Transmission and within-host spread of pathogenic organisms are associated with selective and nonselective bottlenecks that significantly reduced population diversity. In several bacterial pathogens, hypermutable mechanisms have evolved that mediate high-frequency reversible switching of specific phenotypes, such as surface structures, and hence counteract bottleneck-associated reductions in population diversity. Here, we investigated how combinations of hypermutable simple sequence repeats interact with nonselective bottlenecks by using a stochastic computer model and experimental data for *Campylobacter jejuni*, a food-borne pathogen. We find that bottleneck size qualitatively alters the output populations, with large bottlenecks maintaining population diversity while small bottlenecks produce dramatic shifts in the prevalence of particular variants. We conclude that narrow bottlenecks are capable of producing host-to-host variation in repeat-controlled bacterial phenotypes, leading to a potential for stochastic person-to-person variations in disease outcome for *C. jejuni* and other organisms with similar hypermutable mechanisms.

## INTRODUCTION

Many bacterial species exhibit phase variation (PV), defined as high-frequency, reversible, heritable, stochastic switching between phenotypic states (1). The same or similar phenomena are found in the literature under a variety of names, including “stochastic phenotype switching” (2) and “bet-hedging” (3), while the loci themselves are sometimes referred to as “contingency loci” (4).

The food-borne pathogen *Campylobacter jejuni* serves as a model organism for studying phase-variable loci. *C. jejuni* is a flagellated, spirally shaped, Gram-negative bacterium that is the leading cause of gastroenteritis in the developed world (5). In *C. jejuni*, phase-variable switching results from instability in homonucleotide G or C repeats, mostly located within the reading frame of genes. These repeat tracts mutate at 1 × 10^−4^ to 4 × 10^−3^ mutations/division (6), depending on the repeat number, with most indels being of a single nucleotide, resulting in reversible inactivation of gene expression (i.e., an on-off switch). There are 29 of these repeat sequences in the NCTC 11168 strain (7) that influence a variety of functions, including adhesion, aggregation, phage sensitivity, and serum resistance (8–11). One consequence of *C. jejuni* having multiple phase-variable loci is the generation of a large number of combinatoric variations in phenotype. The combined on-off state of different phase-variable loci is referred to as the phasotype (12).

Bacterial populations are subject to frequent bottlenecks due to strong selection acting on specific phenotypes and to nonselective reductions in populations resulting from, for example, a physical disruption of a larger population during transmission. Selective bottlenecks may act on specific loci but will impose severe reductions in the genetic variability of all other phenotypes. These bottlenecks can remove beneficial mutants from the population (13) or preserve “transmission mutants” which are growth deficient but better able to transfer between hosts (14). There is evidence that severe bottlenecks occur during bacterial pathogenesis with evidence from the use of isogenic, tagged mutants that a single cell may be responsible for initiation of an infection following passage between compartments (e.g., nasopharynx to bloodstream) (15, 16). PV may allow bacteria to “eat their cake and have it too” by rapidly switching between growth and transmission states and rapidly reestablishing population diversity following such bottlenecks.

Beaumont et al. (3) demonstrated that repeated selective bottlenecks alternating between selection for one trait and then selection for the absence of the same trait could result in the rapid evolution of bet hedging in *Pseudomonas fluorescens*, in which the bacteria developed rapid switching between an opaque and a translucent colony morphology. Although this switching was later determined to be generated by a form of epigenetic bistability (17), the computer model created to explain the evolution of the phenomena is directly applicable to PV. Thus, Libby and Rainey (2) used a simple *in silico* model of these experimental conditions where each cell was modeled as being in one of two selectively relevant states and either highly mutable or not. Given a small chance for the highly mutable state to emerge from the low-mutability variants and selective bottlenecks applied periodically, the model shows that highly mutable variants arise rapidly and invade populations under a broad range of conditions (2).

Using experimentally derived PV rates for a tetranucleotide repeat tract, Palmer et al. (18) demonstrated that the mutability of a single locus was determined by the combination of strength and period of selection acting on each expression state of the locus. While oscillating locus-specific selection is expected to occur, actual experimental evidence is lacking because of the technical difficulties of generating a suitable model. There is, however, evidence of a selective advantage of high mutation rates during selection for on-to-off switching (19, 20). As these studies indicate, hypermutable loci have a defined, reproducible impact on survival during periodic locus- or phenotype-specific selection. The interplay between one or more hypermutable locus and nonselective reductions in a population is less predictable but of similar importance, given the central role of these types of bottlenecks in bacterial pathogenesis. While changes in levels and combinations of PV expression states have been examined in both model and epidemiological samples, there are significant difficulties with explicit separation of the effects of mutability, selection, and bottlenecks.

In this paper, we present a stochastic simulation of PV under repeated nonselective bottlenecks and compare the results of this simulation to PV data obtained from an experimental model involving nonselective passaging of *C. jejuni* populations through bottlenecks of 1 to >1,000 cells. We find that population behavior under nonselective bottlenecks depends on the size of the bottleneck, with small bottlenecks producing an effect qualitatively different from that produced by large bottlenecks, and the diversity of the initial population. Our models indicate how small nonselective bottlenecks may result in stochastic effects on infection by phase-variable bacterial pathogens and, conversely, how larger bottlenecks may maintain a highly diverse phase-variable population able to rapidly respond to host or environmental selection pressures.

## RESULTS

### Experimental testing of the impact of repeated bottlenecks.

**FIG 1:**
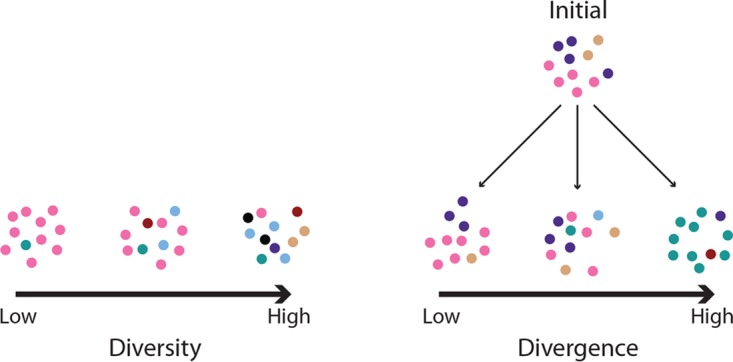
Illustration of the difference between diversity and divergence. Circles represent cells, with different colors corresponding to different phasotypes. On the left are three populations with increasing diversity, going from left to right. The low-diversity population is dominated by a single phasotype, while the high-diversity population contains many phasotypes in roughly equal proportions. This is a within-population measurement, whereas divergence is a between-populations measurement; thus, the three populations shown on the right are increasingly diverged from the “initial” population above, going from left to right. The first population has low divergence because it contains the same phasotypes as the initial population and in similar proportions, while the high-divergence population shares no phasotypes with the initial population. Note that this high-divergence population has low diversity since it is dominated by a single phasotype.

Bottlenecks can constrain and impact the patterns of genetic variation in bacterial populations. To test how nonselective bottlenecks influence the genetic structure of phase-variable gene expression patterns, we subjected *C. jejuni* strain NCTC11168 to multiple passages on Mueller-Hinton agar (MHA) plates and interposed bottlenecks between passages. To assess the effects of different bottleneck sizes on population structure, we calculated diversity and divergence scores for each of the input and output populations (Fig. 1). The former measures the proportions of each combination of phase-variable expression states arising from switching of multiple genes, termed phasotypes, while the latter measures the difference in the phasotype proportions between the initial and output populations.

**FIG 2:**
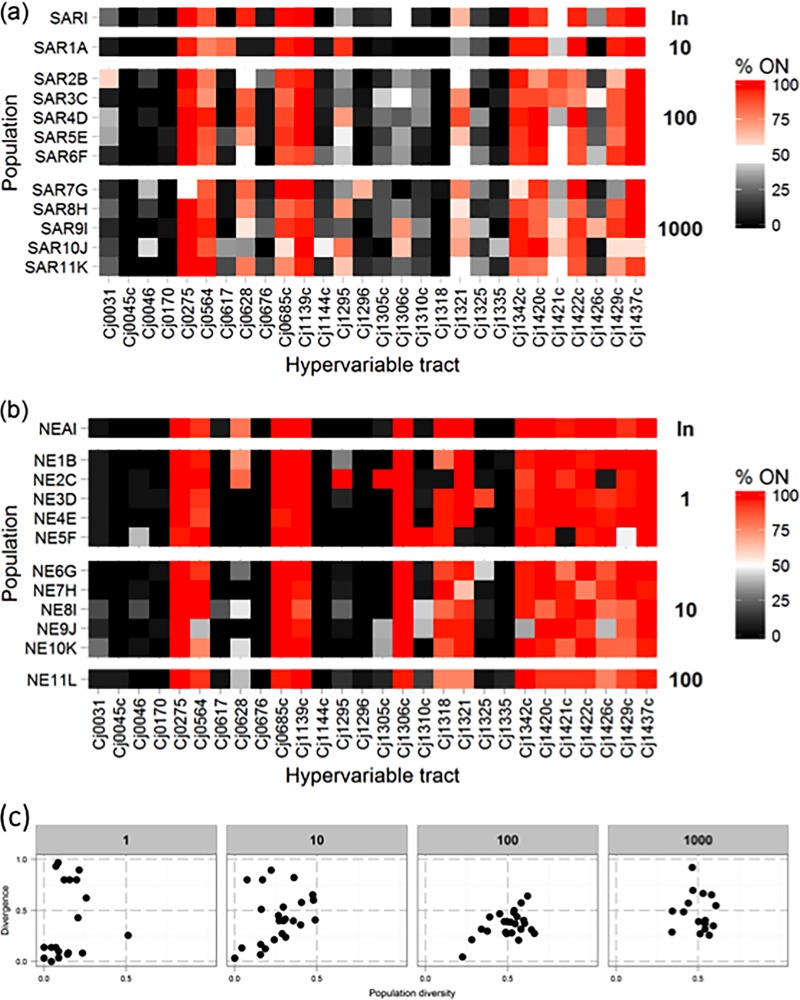
Changes in %on values during an *in vitro* bottleneck experiments with *C. jejuni* strain NCTC11168. Populations of *C. jejuni* were subject to a series of five passages on MHA plates. Bottlenecks were imposed between passages by harvesting colonies from serial dilutions of each population. Samples of 60 or 30 colonies were collected for the inoculum and each lineage, respectively. For every phase-variable gene of each colony, expression states were determined from repeat numbers with the 28-locus PV assay. For each population, the proportion of each gene in the on state was converted to a percentage and plotted on a color scale. Labels to the left indicate individual lineages, and labels to the right indicate the bottleneck size imposed or “In” for the initial inoculum. Panels a and b are for two separate experiments in which all later populations were started with the same inoculum (SARI and NEAI, respectively). Panel c shows a plot of the diversity-versus-divergence scores of four groups of six phase-variable genes (see Table 1) of each of the output populations separated by bottleneck size.

A bottleneck involved the plating of serial dilutions of bacterial suspensions onto MHA plates and random picking of 1 to 1,000 colonies. This procedure should not impose selection on any of the phase-variable loci and should result in each colony initiating from a single cell. Two experiments were performed, each of which started from separate inocula and involved the application of five repeated bottlenecks to one to five separate lineages. A representative sample of 30 to 60 colonies was picked from each inoculum and output population. Repeat numbers were determined for 28 phase-variable loci and utilized for derivation of binary (0, 1) expression states. The expression states of the multiple colonies were converted into a percent on (%on) value for each sample (Fig. 2).

The one-cell bottleneck (Fig. 2B) exhibited major shifts in the %on values of some genes of the output populations compared to the inoculum but with a random pattern for each lineage. For example, lineage NE2C had an on-to-off switch in *cj1318* and *cj1426c* while NE5F has an off-to-on switch in *cj1310* and an on-to-off switch in *cj1421*. Switches in larger bottlenecks tended to be partial changes in proportions with more homogeneity between lineages (Fig. 2A).

The total number of possible phasotypes for 28 phase-variable genes is 2^28^ = 3 × 10^8^. However, our sample sizes (*n* = 30) limits the accurate measurement of diversity statistics to analysis of 32 to 128 phasotypes, depending on the population structure (see Fig. S1 in the supplemental material). Accordingly, we assigned 24 of the tracts to four groups of six genes (Table 1). Four genes were excluded because of incomplete data sets for some samples. These groups were chosen to have approximately equal proportions of genes with different repeat numbers (since this factor is a major determinant of the PV rate) and with a similar distribution throughout the genome. These groups will behave independently if PV operates independently at each locus and the experiment was nonselective.

10.1128/mBio.02311-16.1 FIG S1  Impact of the number of genes on the accuracy of diversity estimates based on 30 samples. Simulated infinite populations of variable diversity were generated by repeatedly applying the expected level of switching to an initially uniform population for four to nine genes with a mutation rate of 1 in every 300 divisions per gene. For each population, a Monte Carlo simulation of the impact of sampling was generated by generating samples with a size of 30 from the population 1,000 times and calculating the mean diversity of these populations. Points show the ratio of the diversity estimated from these samples to the true diversity, and lines show the best fit calculated by the local polynomial regression fitting (known as "LOESS") method. Illustrated is a close match between estimate and true diversity with six genes and a sample size of 30, with most points within 5% of the true value. The exact ratio is highly sensitive to the method used to generate populations (data not shown), but these populations are designed to mimic real populations. Download FIG S1, PDF file, 0.5 MB. Copyright © 2017 Aidley et al. 2017 Aidley et al. This content is distributed under the terms of the Creative Commons Attribution 4.0 International license.

**TABLE 1:** Genic composition of *C. jejuni* phasotypes

Group	Genes (on length)
A	*cj0676* (10), *cj1144c* (10), *cj1296* (10), *cj1310c* (9), *cj1325* (9), *cj1422c* (9)
B	*cj0045c* (11), *cj0617* (10), *cj1139c* (8), *cj1305c* (9), *cj1321* (10), *cj1426c* (10)
C	*cj0170* (8), *cj0564* (10), *cj0685c* (9), *cj1306c* (9), *cj1342c* (9), *cj1429c* (10)
D	*cj0031* (9), *cj0275* (8), *cj0628* (11), *cj1295* (9), *cj1318* (11), *cj1420c* (9)

To visualize the effects of the experimental bottlenecks, we plotted the diversity and divergence values against each other (Fig. 2C). The expected constraint of small bottlenecks on the numbers of variants in a population was apparent, with diversity increasing as a function of bottleneck size. There was, however, a striking effect of bottlenecks on the differences between input and output populations. Single-cell bottleneck populations exhibited a bimodal pattern of either high or low divergence from the input population. Conversely, the majority of output populations for the 1,000-cell bottleneck had intermediate levels of divergence.

### *In silico* simulation of phase-variable populations under repeated bottlenecks.

The findings from our experimental model of bottlenecks were provocative but difficult to explore further because of the difficulties of performing multiple replicates and of the manipulation of key parameters such as the PV rate. We chose, therefore, to develop a simplified *in silico* model that would be applicable to a wider range of bacterial organisms. The model was predicated on our experimental setting and utilized relevant PV rates and population sizes but ignored complexities such as the differences in rates due to on-to-off versus off-to-on switching, various tract lengths, and the variations in poly(G) tract lengths between loci (6). Thus, the switching rates for each locus were reduced to a binary on-off model with symmetrical switching rates for on-to-off and off-to-on directional switches. The other key features of the simulation are that phasotypes were tracked as groups rather than as individual cells, with changes occurring in every generation of a discrete (as opposed to a continuous)-time model.

To capture the dynamics of growth and bottlenecks, the model has a discrete growing population that doubles in size at each generation and has a probability of switching applied at each generation. This switching is applied to the new growth, so one-half of the new generation is identical to the previous generation (this mimics the expectation from slipped-strand mispairing of a simple sequence repeat where the template strand is unaltered while the newly synthesized strand contains an indel). To study the behavior of phasotypes, the model incorporated multiple phase-variable genes, each of which switches independently.

To increase performance, the population was modeled as counts of each phasotype rather than individual cells in a manner mathematically equivalent to the modeling of each cell. To do this, a change of phasotype was generated by picking a random number from a multinomial distribution and using the binary representation of this number to indicate the specific gene that was subject to a change in expression—i.e., 010000 (2 in decimal) represents a change of state in the second gene while 001010 (20 in decimal) represents changes in the third and fifth genes. To exactly simulate independent switching at each of the genes, a number between 0 and 2^*G*^ − 1 (where *G* is in the number of genes modeled, 0 represents no change, and 2^*G*^ − 1 represents change at all loci) was derived from the multinomial distribution with the probability of each number being based on its binary representation. Summing the digits in the binary representation gives the number of loci that will change from the original phasotype, and thus, the probability for each number can be given as *P*_(*n*)_ = *p*^*b*^ for *n* ≠ 0, where *b* is the sum of the digits in the binary representation of *n* and *p* is the probability of switching for each locus. Finally, *P*(0) is simply given as 1 less the sum of *P*_(*n*)_ for all *n* ≠ 0 so that the total probability sums to 1.

Every “generation” of the simulator involves sampling of the multinomial distribution of each phasotype using a sample size equal to the number of cells of that phasotype in the parental population. This process generates the new phasotypes whose numbers are combined with the parental phasotypes to give the total number of cells of each phasotype. The outcome is that the original parental population is doubled with the desired property of an independent probability of switching being applied to every locus in half of the new population.

We selected a maximum population size that approached the expected sizes for our experimental data sets. Thus, growth was allowed to proceed to a limiting size of approximately 10^9^ cells (i.e., 30 generations, with the exact size being dependent on whether the initial population size was a power of 2). Bottlenecks were applied by randomly selecting a number of individuals from the population by using a uniform probability of selection without replacement. This subpopulation was then used to seed the next generation. This bottleneck could be repeated by allowing the population to grow to its maximum size before reapplying the bottleneck. The model was run multiple times with a constant input population. This input population was created by running the model 100 times from a single cell to the maximum population size and then selecting the population with the median diversity as the initial input for the model.

Variation and the application of bottlenecks in this model are fully stochastic, so each run of the model produces a different output and thus the results presented are from multiple runs of the model.

### Effect of *in silico* bottleneck size on population dynamics.

**FIG 3:**
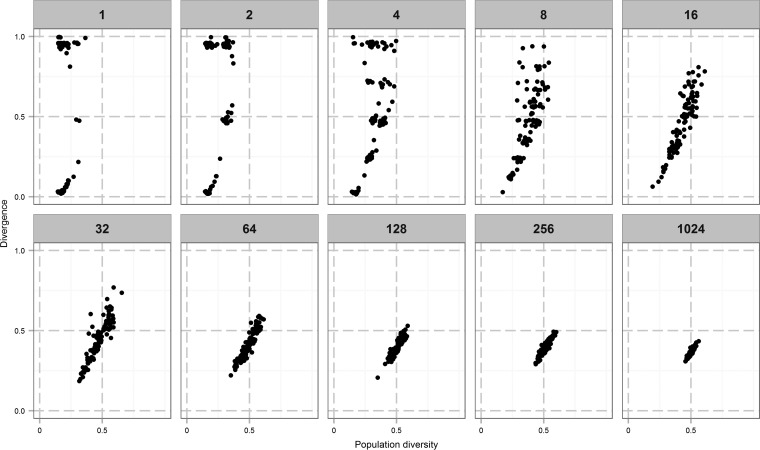
Divergence and diversity in simulated data with a range of bottleneck sizes. Each point represents an individual run of the simulator with 100 runs for each bottleneck size. The model simulates a six-gene phasotype with each gene switching at 0.002 mutation per division for both directions of PV (i.e., on to off and off to on). The starting population has an initial diversity of 0.13. Each population was grown to a size of approximately 10^7^ (24 divisions). Diversity is measured by Shannon equitability, and divergence is the difference from the initial inoculum quantified as population separation.

We modeled the effect of five repeated bottlenecks on virtual populations with six genes and a mutation rate of 1 in 500, which is in the middle of the estimated range of PV rates for *C. jejuni*. After each bottleneck, the population was grown to a size of 2^30^ before the next bottleneck was applied. We ran the simulator 100 times for each bottleneck size and then plotted diversity versus divergence for each of the output populations (Fig. 1). Each point in Fig. 3 is the outcome of one run of the simulator. These data show that bottleneck size produces major quantitative and qualitative shifts in the output populations. Thus, small bottlenecks produced a bimodal pattern with distinct output populations of high and low divergence but both of low diversity. Contrastingly, larger bottlenecks result in convergence on populations with intermediate levels of both divergence and diversity. Divergence is still generated with the larger bottlenecks but is now due to the diversity generated during replication from a population, which becomes more diverse with every replication, rather than from a direct effect of the bottleneck.

**FIG 4:**
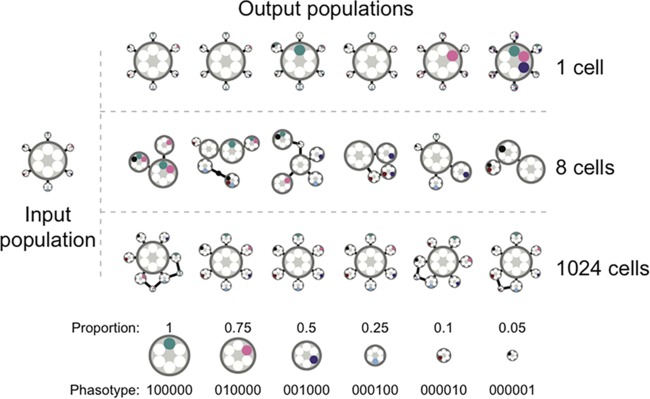
Changes in output population structure after application of bottlenecks. Shown is the population structure from six independent runs of the simulator with three different bottleneck sizes. The common input population is shown on the left. Each population is represented by a group of linked circles where each circle represents a different phasotype with the area of the circle proportional to the proportion of the population in that phasotype. The six inner circles represent the phasotype with filled (colored) circles representing the on state for that gene and empty (white) circles representing the off state. All single-gene changes are linked by solid lines, while those lines with a circle in the middle represent two gene changes and are only included where necessary to produce a connected graph. Phasotypes that compose <1% of the population are omitted. These make up an average of 0.6% of the population for 1-cell bottlenecks, 5.1% for 8-cell bottlenecks, and 7.5% for 1,024-cell bottlenecks.

Bottleneck size also impacts population structure. With single-cell bottlenecks and the larger bottlenecks, the population structure is similar in all output populations, whereas intermediate smaller bottleneck sizes produce disrupted population structures. This is illustrated in Fig. 4, where six example output populations derived from the same input population with 1-, 8-, or 1,024-cell bottlenecks are shown. In the case of 1- and 1,024-cell bottlenecks, the population contains a single dominant phasotype and then a cluster of phasotypes closely derived from this phasotype, whereas in the 8-cell bottleneck, the population structure is disrupted and contains multiple phasotypes that each contribute a large proportion of the population. In the case of the 1,024-cell bottleneck, the major phasotype is always the same major phasotype as that present in the input population; contrastingly, roughly half of the output populations from the single-cell bottleneck have a new major phasotype. Critically, this new major phasotype is not only novel with respect to the input population but differs between output populations. This new major phasotype also makes up a large proportion (>85%) of the population under these single-cell conditions. Rare phasotypes (those where the individual phasotype composes <1% of the population) also make up an increasing proportion of the total population as the bottleneck size increases (0.6% of the population for 1-cell bottlenecks, 5.1% for 8-cell bottlenecks, and 7.5% for 1,024-cell bottlenecks).

In some cases, the significant factor may not be the diversity within the population but simply the presence or absence of particular phasotypes. To investigate the chances of a particular phasotype being generated, the coverage of the space of all possible phasotypes was calculated with a range of bottleneck sizes and simulated generations (Table 2). With a large final population (2^30^), the mean coverage was 62.80 phasotypes even in the single-cell bottleneck—or almost complete coverage of the 64 possible phasotypes with six genes—while a smaller final population (2^24^) produced a mean coverage of 50.54 phasotypes in a single bottleneck and 62.24 with a 1,024-cell bottleneck. Note that the coverage is not uniform, with the phasotypes most distant from the starting phasotype being less likely to be reached. These changes in final population size did not, however, materially alter the diversity or divergence of these simulations (data not shown).

**TABLE 2:** Coverage of the phasotype space changes with bottleneck size^a^

Bottleneck size	% of populations with all possible phasotypes (mean) for populations with a final size of:		
	2^24^	2^27^	2^30^
1	0 (50.54)	0 (58.55)	15 (62.80)
2	0 (52.27)	6 (60.35)	37 (63.26)
8	2 (58.04)	26 (62.30)	84 (63.83)
128	5 (60.18)	51 (63.40)	97 (63.97)
1,024	11 (62.24)	87 (63.86)	100 (64.00)

a Shown are the mean number of phasotypes present in the final population from 100 runs of the simulator and the percentage of those runs in which all possible phasotypes (*n* = 64) were produced in the final population. Because the simulator is stochastic, repeating the sample of a hundred runs of the simulator will give a different percentage of runs, resulting in total coverage and a slightly different mean. The values shown are representative.

### Varying the number of genes and mutation rate has a limited effect on the impact of bottleneck size.

As there are significant variations in both the mutability and number of phase-variable genes within and between *C. jejuni* genomes, we ran the model with a series of mutation rates ranging from 1 in 200 to 1 in 1,500 mutations per division and between 3 and 10 genes. These parameters had only minor effects on the qualitative properties of the output populations (see Fig. S2 and S3, respectively). Quantitative effects were observed, with higher numbers of genes increasing the divergence produced by smaller bottlenecks. Similarly, and as expected, higher mutation rates increased both the diversity and divergence across all bottleneck sizes. Opposite effects were produced by smaller numbers of genes or lower mutation rates.

10.1128/mBio.02311-16.2 FIG S2  Impact of the mutation rate on simulated populations. Each point represents a single run of the simulation, which was run 50 times for each combination of bottleneck size and mutation rate. Bottleneck size is shown at the top, increasing from left to right, while the mutation rate is shown on the right, decreasing from top to bottom. Download FIG S2, PDF file, 0.6 MB. Copyright © 2017 Aidley et al. 2017 Aidley et al. This content is distributed under the terms of the Creative Commons Attribution 4.0 International license.

10.1128/mBio.02311-16.3 FIG S3  Impact of the number of genes on simulated populations. Each point represents a single run of the simulation, which was run 50 times for each combination of bottleneck size and number of genes. Bottleneck size is shown at the top, increasing from left to right, while the number of genes is shown on the right, increasing from top to bottom. Download FIG S3, PDF file, 0.6 MB. Copyright © 2017 Aidley et al. 2017 Aidley et al. This content is distributed under the terms of the Creative Commons Attribution 4.0 International license.

### Changing the initial population structure impacts the effect of population bottlenecks.

**FIG 5:**
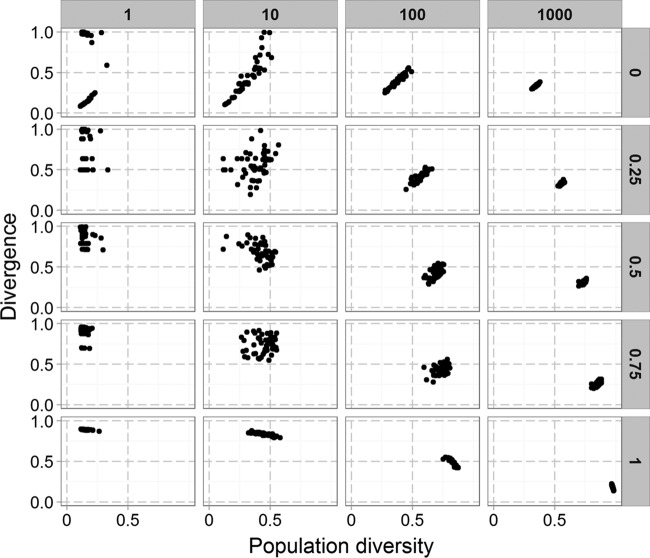
The impact of bottlenecks depends on the initial population structure. Each point represents a single run of the simulation that was run 50 times for each combination of initial population diversity and bottleneck size. Other parameters were as set in Fig. 1. Bottleneck size is shown at the top, increasing from left to right, while initial population diversity is shown on the right, increasing from top to bottom.

The simulations presented in the previous section used a starting population that represented a typical population arising from a single cell and thus mimicked our experimental data sets. However, natural populations exhibit a wide range of diversities. To examine the impact of initial population diversity, we ran the simulator with several different starting populations (Fig. 5). The output populations of the 1,000-cell bottleneck displayed decreasing divergence but increasing diversity as the diversity of the initial population was increased. A similar, but less pronounced, pattern is observed with the 100-cell bottleneck, but with the 10- and 1-cell bottlenecks, the more diverse starting populations only increased divergence but not diversity. This is due to these small bottlenecks restricting the amount of diversity that can pass from one passage to the next. Strikingly, with a maximally diverse population, the bimodal distribution seen with small bottlenecks disappeared and all populations exhibited the maximum divergence from the input population. There was also high divergence between output populations.

### Comparison of experimental and *in silico* phasotype patterns.

To make the *in silico* model as close as possible to the experimental model, the initial population for the simulated runs was seeded with the average proportions from the inoculums used in the experimental data and then grown to the maximum population size before the first bottleneck was applied. A mutation rate of 1 in 300 was chosen as approximating the expected average mutation rate of the actual loci, and populations were grown to a virtual size of approximately 10^7^ cells.

**FIG 6:**
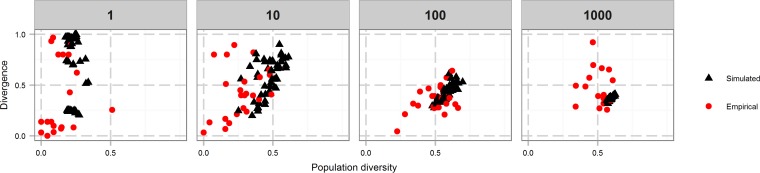
Comparison of empirical and simulated results. Each black triangle represents a separate run of the simulator, while each red dot shows the results generated by grouping genes from the empirical populations calculated from 16 to 30 picked colonies. The values at the top are the sizes of the bottlenecks applied. One lineage, SAR11K (1,000-cell bottleneck), was omitted because of poor data quality.

Divergence and diversity values were calculated separately for each subphasotype in the experimental data and then compared to the output of the model (Fig. 6). The data for the small bottlenecks were similar to the model outputs but increasingly dissimilar for the larger bottlenecks. The experimental data for the 10-cell bottleneck exhibited lower-than-expected diversity for most of the populations, while 40% of the output populations of the 1,000-cell bottleneck had higher-than-expected divergence. To determine whether these differences might result from a systematic effect of sampling from the populations, we ran the *in silico* model with random sampling from the output population and compared the diversity and divergence values obtained from these samples to the true population diversity and divergence values. No systematic bias in the sample sizes used in the *in vivo* experiments was detected (data not shown).

## DISCUSSION

Localized hypermutation—as exemplified by repeat-mediated PV—is a phenomenon exhibited by a diverse range of bacterial pathogens and commensals that has the potential to regenerate genetic diversity of key mediators of host-pathogen interactions following a reduction in population size. Pathogenic bacteria are subject to severe bottlenecks during both spread within and transmission between hosts (15, 21, 22). One outcome of these bottlenecks is a reduction in the amount of genetic variation in the surviving population, which can then limit adaptive potential and propagation as the population is exposed to subsequent selective pressures. Another related but subtle effect is that bottlenecks will generate differences between populations.

### Single-cell bottlenecks as potentiators of disease heterogeneity.

We have observed that single-cell bottlenecks produce a distinctive bimodal distribution in population divergence of phasotypes that is absent in larger bottlenecks. Intermediate bottlenecks show decreasing levels of finer structure as the distributions converge toward the patterns of the larger bottlenecks. This bimodal pattern likely results from rare phasotypes being included in the bottlenecked population, which then come to dominate the new population. This results in a dramatically different population structure in a close parallel to the founder effect in classical evolutionary models (23). Further examination of the diverged populations in single-cell bottlenecks indicated that there was divergence from the initial population but also from each other. A total of 60 divergent populations were found in the 100 *in silico* populations, which was mirrored by 8 of the 20 experimental populations. Each of these diverged populations is dominated by a phasotype with differences in the expression state of one or more genes. After each single-cell bottleneck and growth cycle, there will be loss of populations dominated by the original major phasotype, as between 40 and 60% of the populations adopt a new major phasotype after five cycles in our experiments. This means that the bimodality we observed will decay as a function of the number of bottlenecks applied to the population, with the proportion in the original major phasotype approximately halving every five cycles and a proportionate increase in populations with a diverged major phasotype. Thus, the imposition of a higher number of single-cell bottlenecks will further increase the divergence between populations.

**FIG 7:**
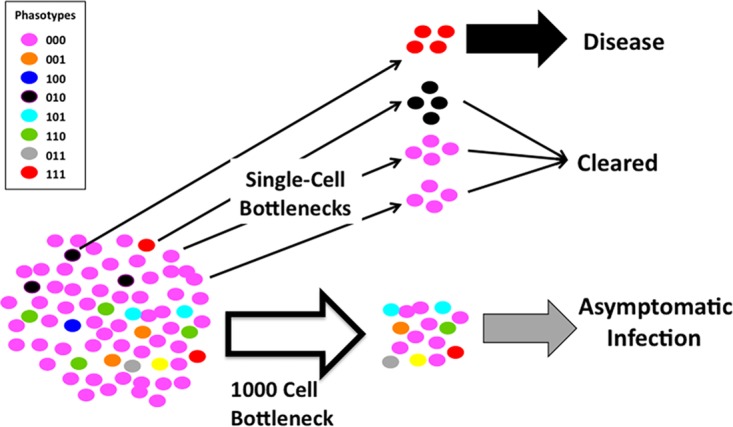
Stochastic impact of nonselective bottlenecks and phase-variable expression states on disease during host-to-host spread. This diagram depicts the potential effects of nonselective bottlenecks altering the population structure of a bacterial species with three phase-variable genes (each capable of switching between the on [1] and off [0] expression states) and hence eight phasotypes (see inset). The initial population from a transmitter host is depicted as having the majority of cells with one combination of expression states (the major “phasotype”) and a subset of six other minor phasotypes. Nonselective bottlenecks, such as the physical reduction in population size that may occur during transmission between hosts, are imposed on this initial population. Single-cell bottlenecks generate a bimodal distribution of output populations either retaining the major phasotype or being dominated by one of the minor phasotypes. One of these phasotypes has the potential to cause disease in a new host, while the others are cleared, resulting in a stochastic effect on the occurrence of disease, with, in the example shown, only one of four hosts exhibiting disease. In the case of a larger 1,000-cell bottleneck, the diversity of the initial population is retained, enabling the population to rapidly establish an asymptomatic infection.

The high levels of divergence caused by single-cell bottlenecks has the potential to impart stochastic effects on the outcome of infections. If specific phasotypes are responsible for disease progression, then only populations where these phasotypes are produced as the dominant type may cause disease (Fig. 7). The net result would be a stochastic appearance of disease in a population even though the pathogenic organism is widely prevalent. High carriage-to-disease ratios are features of meningitis due to *Haemophilus influenzae* and *Neisseria meningitidis* (24–26), two pathogens that contain multiple phase-variable genes with known or putative roles in disease-related phenotypes such as immune evasion (4). Similarly, there are frequent infections with *C. jejuni* strains capable of expressing epitopes responsible for inducing the autoantibodies responsible for neuropathies and yet these postinfection sequelae are rare. These pathogens are subject to small bottlenecks due to passage between compartments (e.g., nasopharynx to bloodstream) or during transmission (e.g., infected chicken through the food chain to contaminated food product). Divergence in phasotypes (i.e., phase-variable gene expression patterns) due to small bottlenecks may be a component of the low disease frequencies of such pathogenic bacteria.

### Maintenance of population divergence over time.

A key facet of PV is that the high mutation rates will counteract the effects of bottlenecks. Using experimentally derived PV rates for *C. jejuni* genes combined with a mathematical analysis of phase-variable populations, Bayliss et al. (6) showed that the rate of approach to a steady state for a particular combination of on-off expression states is dependent on the number of genes and the slowest switching rate. This process will disrupt the divergence introduced by single-cell bottlenecks, as the populations will start to revert to the steady state and hence there will be a temporal limit on the diversifying effects of such bottlenecks. However, the rate of approach to the steady state operates over hundreds of generations and can be prevented by the imposition of selection or additional bottlenecks, as may happen if a population initiates disease or enters a new host compartment.

### Maintenance of population diversity through relatively small bottlenecks.

While very small bottlenecks have a dramatic reductive effect on phasotype population diversity, larger bottlenecks preserve much of the phasotype diversity within a population. This effect begins to dominate even at relatively small bottleneck sizes numbering in the hundreds of cells and is also observed even with highly diverse starting populations. Because each bottleneck preserves the diversity generated during the growth phase of the population, there is a correlation between divergence and diversity as the population diverges from the original by becoming more diverse. This preservation of initial diversity highlights another alternate pathway whereby PV could contribute to disease or host adaptation by a bacterial pathogen (Fig. 7). In situations where the transmitted population is in the range of 100 to 1,000 cells, the diversity generated in one host individual or compartment would be maintained through the transmission event. This preservation of diversity may be important, as it may facilitate adaptation in the new environment. Thus, for example, the phase-variable genes of a *C. jejuni* strain may have reached the optimum fitness state in the cecum of one chicken and preservation of this state through a transmission bottleneck will then facilitate rapid colonization of the next chicken and hence lead to rapid spread through a flock.

### Underlying assumption and difficulties with modeling of experimental outputs.

Our model was not fitted to experimental data by a reiterative series of parameter modifications but rather predicated on a simplified version of known *C. jejuni* PV and growth parameters. This simplified model is therefore applicable to other related systems where localized hypermutation contributes to phenotypic diversity. In particular, we used population sizes, binary division, mutations in only one daughter cell, and mutation rates that are directly relevant to other bacterial species with multiple phase-variable genes. Two simplifying assumptions were that division was synchronous and that PV rates were equal for all loci and for both switching directions. Despite these simplifying assumptions, the major features of the experimental data for bottlenecks with sizes of 1 to 100 cells were reproduced in the *in silico* model, suggesting that this model captured the underlying behavior of the biological system. At the 1,000-cell bottleneck, the correlation between experimental and *in silico* data was weaker than with small bottlenecks, mainly because of greater-than-predicted divergence. Selection does not seem to be responsible for this effect, as there were no specific genic or phasotype differences. One alternative cause is the assumption of identical PV rates for all loci. Minor variations in the mutation rates and directions of switching due to these assumptions may generate minor alterations in population structure that accumulate in large bottlenecks but are disrupted by the stronger effects of small bottlenecks. Improved experimental evaluation of switching rates may be required to further dissect the interplay among the mutability of PV genes, bottlenecks, and population structure.

### Conclusions.

Bottlenecks of various sizes are a significant component of the life cycle of pathogenic bacteria. Our data indicate that while PV can increase or maintain levels of population variation in the face of bottlenecks, smaller bottlenecks may produce host-to-host variation in the prevalence of specific phasotypes. There are two contrasting implications for bacterial disease of these findings, maintenance of an adaptive or disease-causing state as the pathogen passes through a series of bottlenecks or, conversely, host-to-host or compartmental variation in exposure to specific phasotypes leading to disease heterogeneity.

## MATERIALS AND METHODS

### *In silico* modeling.

Modeling was carried out in Python 3.3 (http://www.python.org) by using NumPy 1.9.3 to generate random numbers (27). The model used and the data generated to produce the figures in this paper are available from Dryad (https://doi.org/10.5061/dryad.p46n0).

### Quantification of changes in population structure.

Each *in silico* or *in vitro* experiment produced a population containing various proportions of different six-gene phasotypes. Changes in the phasotypes of each population were quantified along two axes, (i) the number and proportions of different phasotypes within the population, which is referred to as diversity, and (ii) the difference between the input and output populations, which is referred to as divergence. The difference between these measures is illustrated in Fig. 1.

“Diversity” was quantified by using Shannon equitability, which is simply Shannon entropy (also known as the Shannon index) (28) normalized by its maximum possible for the data set to give a number between 0 and 1, where 0 is the minimum possible diversity (the entire population has a single phasotype) and 1 is the maximum possible diversity (all possible phasotypes are present in equal amounts).

“Divergence” was quantified by using population separation, which is simply the proportion of each population not shared by the two populations. This gives a number between 0 (the populations have exactly the same phasotypes present in exactly the same proportions) and 1 (the populations have no phasotypes in common).

Mathematically, these are expressed as follows: *Diversity* = *S*/*S*_max_, where

$$S=-\sum^{\;}\log_{2}p_{i}{}^{p_{i}}$$

where *p*_*i*_ is the proportion of the population in each phasotype and *S*_*max*_ is the maximum value of *S* in this data set. This is achieved when the phasotypes are present in equal quantities and so if the sample or population size, *N*, is less than the number of phasotypes (2^*G*^, where *G* is the number of genes) this is

$$S_{\max}=-\overset{N}{\underset{1}{\sum }}\frac{1}{N}\log_{2}\frac{1}{N}=\log_{2}N$$

or if the number of phasotypes is less than or equal to the sample or population size,

$$S_{\max}=-\overset{2^{G}}{\underset{1}{\sum }}\frac{1}{2^{G}}\log_{2}\frac{1}{2^{G}}=\log_{2}2^{G}=G$$

Divergence is most easily calculated by calculating the population overlap and then subtracting this from 1 to give population separation. This is calculated as follows:

$$Divergence=1-\sum^{\;}min\left(p_{i},q_{i}\right)$$

where *p*_*i*_ and *q*_*i*_ are the phasotype proportions for each population.

### Population bottleneck experiments.

*C. jejuni* NCTC11168 was used in all experiments and was grown under microaerophilic conditions (4% O_2_, 10% CO_2_, 86% N_2_) at 42°C on Müller-Hinton agar (Oxoid) plates supplemented with vancomycin (10 µg/ml) and trimethoprim (5 µg/ml). The initial inoculum for the experiments was prepared by taking the bacteria from frozen glycerol stocks stored at −80°C and then restreaking them onto a second plate, which was then scraped into Müller-Hinton broth (Oxoid) to resuspend the cells.

To apply a population bottleneck, the required number of colonies was picked with a loop (for smaller bottleneck sizes) or estimated and then scraped off the plate (for larger bottleneck sizes) before being resuspended in Müller-Hinton broth and then serially diluted onto agar plates. Individual colonies were picked from the inoculum (60 colonies) and the final population (30 colonies) after five successive bottlenecks. The repeat numbers, expression states, and phasotypes for 28 phase-variable loci of these colonies were determined with the 28-locus PV assay (29).

### Preparation of figures.

Figures were plotted in R 3.2.3 (30) with the ggplot2 (31) and PlyR (32) packages. Color figures were checked for suitability for colorblind readers by using ColorOracle (33).

## References

[1] Lin WH, Kussell E 2012 Evolutionary pressures on simple sequence repeats in prokaryotic coding regions. Nucleic Acids Res 40:2399–2413. doi:10.1093/nar/gkr1078. 22123746 PMC3315296

[2] Libby E, Rainey PB 2011 Exclusion rules, bottlenecks and the evolution of stochastic phenotype switching. Proc Biol Sci 278:3574–3583. doi:10.1098/rspb.2011.0146. 21490013 PMC3189364

[3] Beaumont HJE, Gallie J, Kost C, Ferguson GC, Rainey PB 2009 Experimental evolution of bet hedging. Nature 462:90–93. doi:10.1038/nature08504. 19890329

[4] Moxon R, Bayliss CD, Hood D 2006 Bacterial contingency loci: the role of simple sequence DNA repeats in bacterial adaptation. Annu Rev Genet 40:307–333. doi:10.1146/annurev.genet.40.110405.090442. 17094739

[5] Young KT, Davis LM, Dirita VJ 2007 Campylobacter jejuni: molecular biology and pathogenesis. Nat Rev Microbiol 5:665–679. doi:10.1038/nrmicro1718. 17703225

[6] Bayliss CD, Bidmos FA, Anjum A, Manchev VT, Richards RL, Grossier JP, Wooldridge KG, Ketley JM, Barrow PA, Jones MA, Tretyakov MV 2012 Phase variable genes of Campylobacter jejuni exhibit high mutation rates and specific mutational patterns but mutability is not the major determinant of population structure during host colonization. Nucleic Acids Res 40:5876–5889. doi:10.1093/nar/gks246. 22434884 PMC3401435

[7] Parkhill J, Wren BW, Mungall K, Ketley JM, Churcher C, Basham D, Chillingworth T, Davies RM, Feltwell T, Holroyd S, Jagels K, Karlyshev AV, Moule S, Pallen MJ, Penn CW, Quail MA, Rajandream MA, Rutherford KM, van Vliet AH, Whitehead S, Barrell BG 2000 The genome sequence of the food-borne pathogen Campylobacter jejuni reveals hypervariable sequences. Nature 403:665–668. doi:10.1038/35001088. 10688204

[8] Ashgar SSA, Oldfield NJ, Wooldridge KG, Jones MA, Irving GJ, Turner DPJ, Ala’Aldeen DAA 2007 CapA, an autotransporter protein of Campylobacter jejuni, mediates association with human epithelial cells and colonization of the chicken gut. J Bacteriol 189:1856–1865. doi:10.1128/JB.01427-06. 17172331 PMC1855769

[9] Karlyshev AV, Linton D, Gregson NA, Wren BW 2002 A novel paralogous gene family involved in phase-variable flagella-mediated motility in Campylobacter jejuni. Microbiology 148:473–480. doi:10.1099/00221287-148-2-473. 11832511

[10] Holst Sørensen MCH, van Alphen LB, Fodor C, Crowley SM, Christensen BB, Szymanski CM, Brøndsted L 2012 Phase variable expression of capsular polysaccharide modifications allows Campylobacter jejuni to avoid bacteriophage infection in chickens. Front Cell Infect Microbiol 2:11. doi:10.3389/fcimb.2012.00011. 22919603 PMC3417653

[11] van Alphen LB, Wenzel CQ, Richards MR, Fodor C, Ashmus RA, Stahl M, Karlyshev AV, Wren BW, Stintzi A, Miller WG, Lowary TL, Szymanski CM 2014 Biological roles of the O-methyl phosphoramidate capsule modification in Campylobacter jejuni. PLoS One 9:e87051. doi:10.1371/journal.pone.0087051. 24498018 PMC3907429

[12] Alamro M, Bidmos FA, Chan H, Oldfield NJ, Newton E, Bai X, Aidley J, Care R, Mattick C, Turner DPJ, Neal KR, Ala’aldeen DAA, Feavers I, Borrow R, Bayliss CD 2014 Phase variation mediates reductions in expression of surface proteins during persistent meningococcal carriage. Infect Immun 82:2472–2484. doi:10.1128/IAI.01521-14. 24686058 PMC4019173

[13] Wahl LM, Gerrish PJ, Saika-Voivod I 2002 Evaluating the impact of population bottlenecks in experimental evolution. Genetics 162:961–971. 12399403 10.1093/genetics/162.2.961 PMC1462272

[14] Handel A, Bennett MR 2008 Surviving the bottleneck: transmission mutants and the evolution of microbial populations. Genetics 180:2193–2200. doi:10.1534/genetics.108.093013. 18854584 PMC2600951

[15] Gerlini A, Colomba L, Furi L, Braccini T, Manso AS, Pammolli A, Wang B, Vivi A, Tassini M, van Rooijen N, Pozzi G, Ricci S, Andrew PW, Koedel U, Moxon ER, Oggioni MR 2014 The role of host and microbial factors in the pathogenesis of pneumococcal bacteraemia arising from a single bacterial cell bottleneck. PLoS Pathog 10:e1004026. doi:10.1371/journal.ppat.1004026. 24651834 PMC3961388

[16] Moxon ER, Murphy PA 1978 Haemophilus influenzae bacteremia and meningitis resulting from survival of a single organism. Proc Natl Acad Sci U S A 75:1534–1536. doi:10.1073/pnas.75.3.1534. 306628 PMC411507

[17] Gallie J, Libby E, Bertels F, Remigi P, Jendresen CB, Ferguson GC, Desprat N, Buffing MF, Sauer U, Beaumont HJE, Martinussen J, Kilstrup M, Rainey PB 2015 Bistability in a metabolic network underpins the de novo evolution of colony switching in Pseudomonas fluorescens. PLoS Biol 13:e1002109. doi:10.1371/journal.pbio.1002109. 25763575 PMC4357382

[18] Palmer ME, Lipsitch M, Moxon ER, Bayliss CD 2013 Broad conditions favor the evolution of phase-variable loci. mBio 4:e00430-12. doi:10.1128/mBio.00430-12. PMC3546556 23300246

[19] Bayliss CD, Hoe JC, Makepeace K, Martin P, Hood DW, Moxon ER 2008 Neisseria meningitidis escape from the bactericidal activity of a monoclonal antibody is mediated by phase variation of lgtG and enhanced by a mutator phenotype. Infect Immun 76:5038–5048. doi:10.1128/IAI.00395-08. 18694967 PMC2573340

[20] Tauseef I, Ali YM, Bayliss CD 2013 Phase variation of PorA, a major outer membrane protein, mediates escape of bactericidal antibodies by Neisseria meningitidis. Infect Immun 81:1374–1380. doi:10.1128/IAI.01358-12. 23403557 PMC3639595

[21] Meynell GG, Maw J 1968 Evidence for a two-stage model of microbial infection. J Hyg (Lond) 66:273–280. doi:10.1017/S0022172400041139. 4885483 PMC2130631

[22] Rubin LG 1987 Bacterial colonization and infection resulting from multiplication of a single organism. Rev Infect Dis 9:488–493. doi:10.1093/clinids/9.3.488. 3299635

[23] Barton NH, Charlesworth B 1984 Genetic revolutions, founder effects, and speciation. Annu Rev Ecol Syst 15:133–164. doi:10.1146/annurev.es.15.110184.001025.

[24] Coen PG, Heath PT, Barbour ML, Garnett GP 1998 Mathematical models of Haemophilus influenzae type b. Epidemiol Infect 120:281–295. doi:10.1017/S0950268898008784. 9692607 PMC2809406

[25] García-Rodríguez JA, Fresnadillo Martínez MJ 2002 Dynamics of nasopharyngeal colonization by potential respiratory pathogens. J Antimicrob Chemother 50(Suppl S2):59–73. doi:10.1093/jac/dkf506. 12556435

[26] Caugant DA, Tzanakaki G, Kriz P 2007 Lessons from meningococcal carriage studies. FEMS Microbiol Rev 31:52–63. doi:10.1111/j.1574-6976.2006.00052.x. 17233635

[27] Van Der Walt S, Colbert SC, Varoquaux G 2011 The NumPy array: a structure for efficient numerical computation. Comput Sci Eng 13:22–30. doi:10.1109/MCSE.2011.37.

[28] Shannon CE 1948 A mathematical theory of communication. Bell Syst Tech J 27:379–423. doi:10.1002/j.1538-7305.1948.tb01338.x.

[29] Lango-Scholey L, Aidley J, Woodacre A, Jones MA, Bayliss CD 2016 High throughput method for analysis of repeat number for 28 phase variable loci of Campylobacter jejuni strain NCTC 11168. PLoS One 11:e0159634. doi:10.1371/journal.pone.0159634. 27466808 PMC4965091

[30] R Core Team 2016 R: a language and environment for statistical computing. R Foundation for Statistical Computing, Vienna, Austria.

[31] Wickham H 2009 ggplot2: elegant graphics for data analysis. Springer, New York, NY.

[32] Wickham H 2011 The split-apply-combine strategy for data analysis. J Stat Softw 40:1–29.

[33] Jenny B, Kelso NV 2007 Color design for the color vision impaired. Cartogr Perspect 57:61–67. doi:10.14714/CP58.270.

