# Erratum for Aidley et al., “Nonselective Bottlenecks Control the Divergence and Diversification of Phase-Variable Bacterial Populations”

**DOI:** 10.1128/mBio.00878-17

**Published:** 2017-08-08

**Authors:** Jack Aidley, Shweta Rajopadhye, Nwanekka M. Akinyemi, Lea Lango-Scholey, Michael A. Jones, Christopher D. Bayliss

**Affiliations:** a Department of Genetics, University of Leicester, Leicester, United Kingdom; b School of Veterinary Medicine, University of Nottingham, Nottingham, United Kingdom

## ERRATUM

Volume 8, no. 2, e02311-16, 2017, https://doi.org/10.1128/mBio.02311-16. The byline and affiliation line of our article should appear as shown above. The following should also be added to the end of Acknowledgments: “M.A.J. was supported by the BBSRC (grant BBI02542).”

