# Supplementary figures and images for: Nonselective Bottlenecks Control the Divergence and Diversification of Phase-Variable Bacterial Populations
Source: mBio. 2017 Apr 4;8(2):e02311-16. doi: 10.1128/mBio.02311-16 (PMC5380846; doi:10.1128/mBio.02311-16)

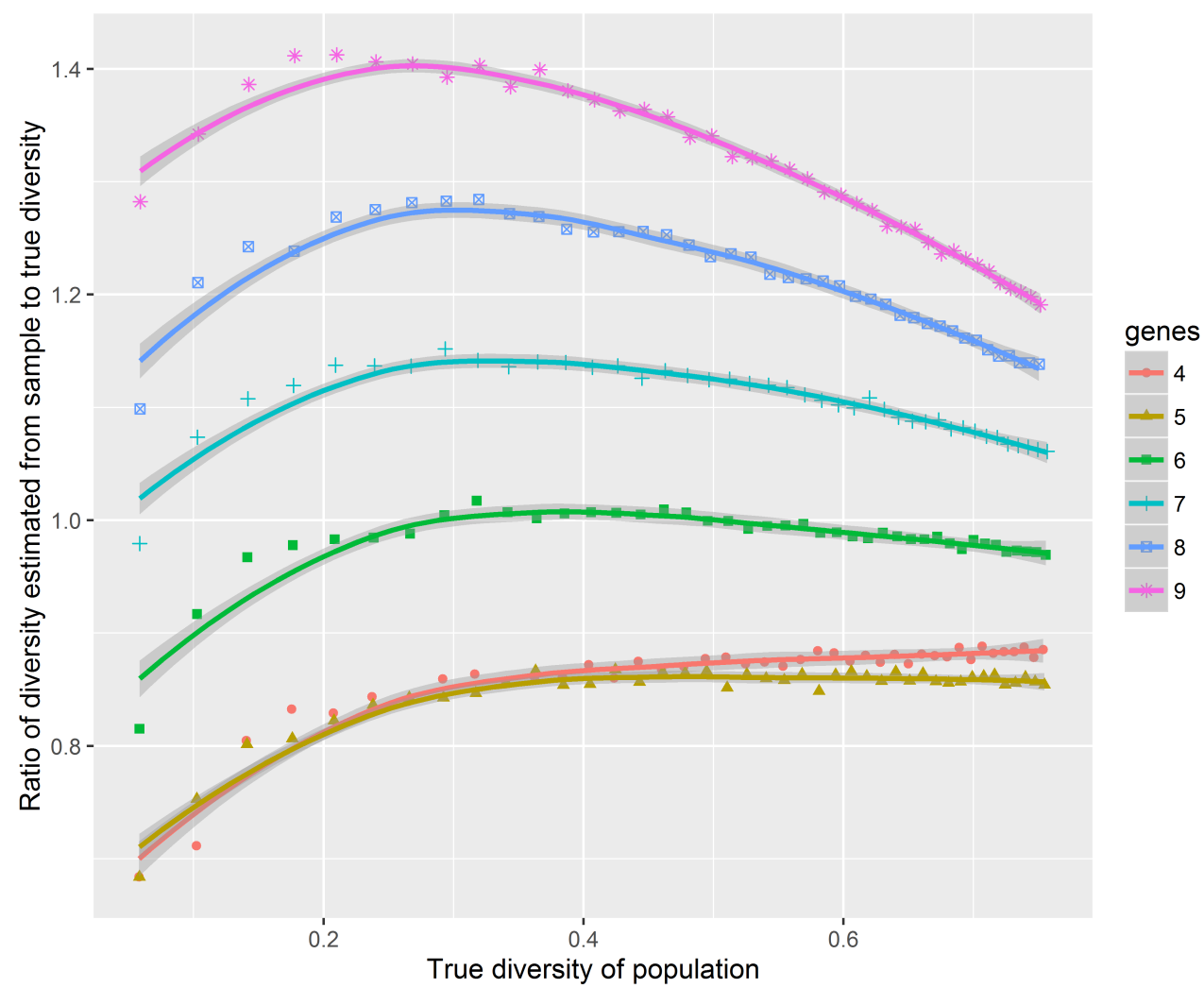

Supplement: FIG S1 [file mbo002173265sf1.pdf]

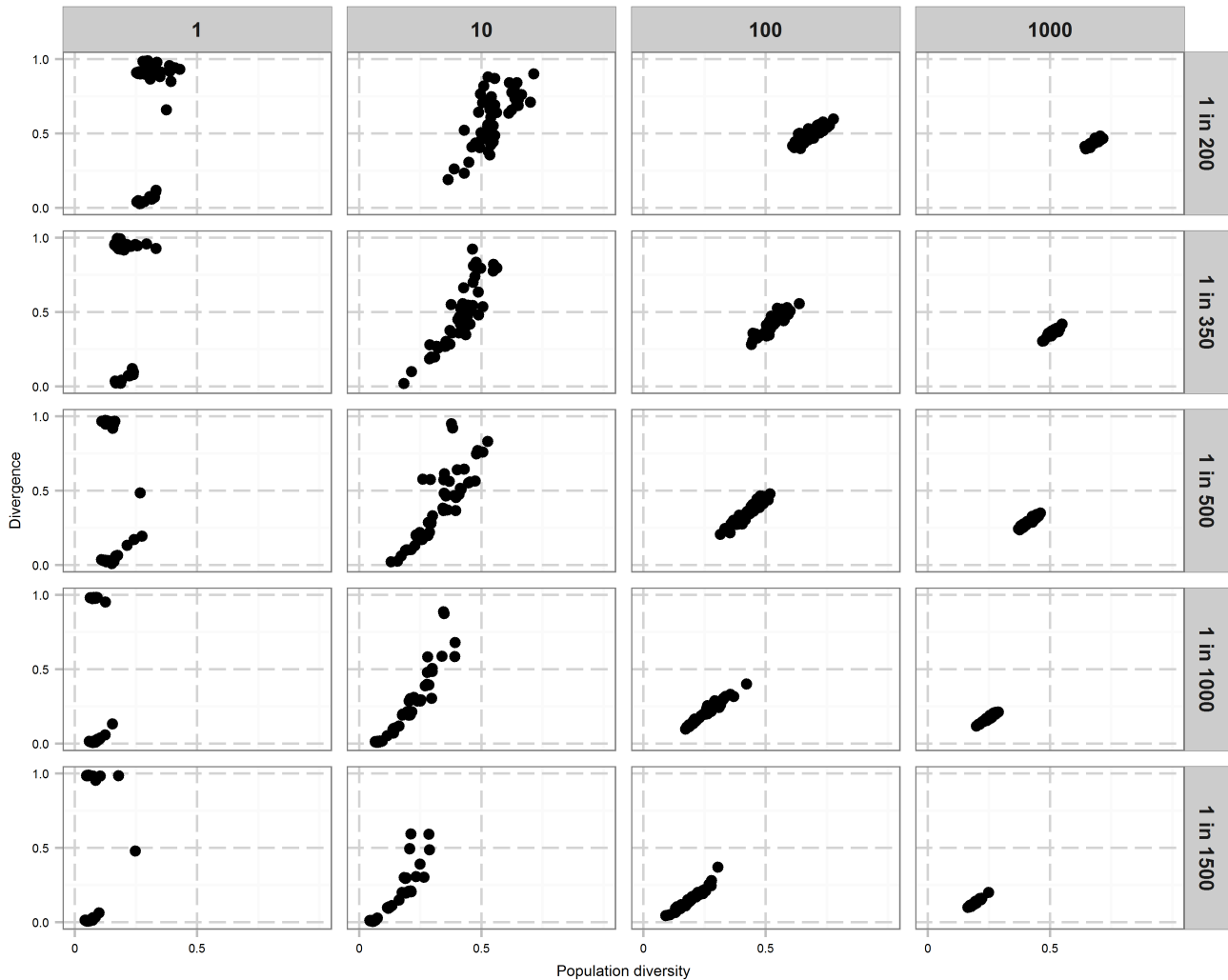

Supplement: FIG S2 [file mbo002173265sf2.pdf]

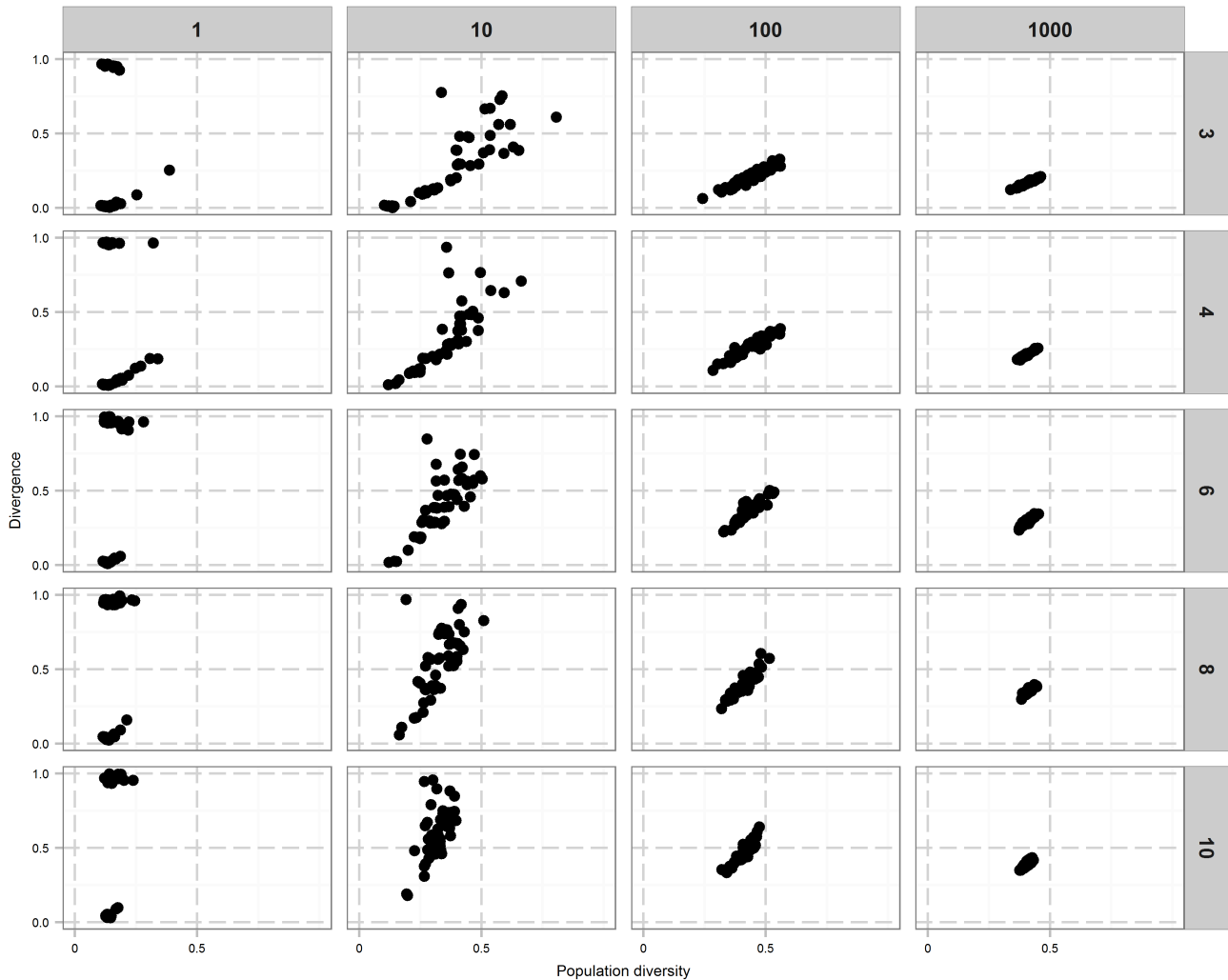

Supplement: FIG S3 [file mbo002173265sf3.pdf]
